# A unified model of ketamine’s dissociative and psychedelic properties

**DOI:** 10.1177/02698811221140011

**Published:** 2022-12-17

**Authors:** Miriam Marguilho, Inês Figueiredo, Pedro Castro-Rodrigues

**Affiliations:** 1 Centro Hospitalar Psiquiátrico de Lisboa, Lisbon, Portugal; 2 Hospital Fernando Fonseca, Amadora, Portugal; 3 NOVA Medical School, NMS, Universidade Nova de Lisboa, Lisbon, Portugal

**Keywords:** Bayesian brain, depression, ketamine, psychedelics

## Abstract

Ketamine is an N-methyl-d-aspartate antagonist which is increasingly being researched and used as a treatment for depression. In low doses, it can cause a transitory modification in consciousness which was classically labelled as ‘dissociation’. However, ketamine is also commonly classified as an atypical psychedelic and it has been recently reported that ego dissolution experiences during ketamine administration are associated with greater antidepressant response. Neuroimaging studies have highlighted several similarities between the effects of ketamine and those of serotonergic psychedelics in the brain; however, no unified account has been proposed for ketamine’s multi-level effects – from molecular to network and psychological levels. Here, we propose that the fast, albeit transient, antidepressant effects observed after ketamine infusions are mainly driven by its acute modulation of reward circuits and sub-acute increase in neuroplasticity, while its dissociative and psychedelic properties are driven by dose- and context-dependent disruption of large-scale functional networks. Computationally, as nodes of the salience network (SN) represent high-level priors about the body (‘minimal’ self) and nodes of the default-mode network (DMN) represent the highest-level priors about narrative self-experience (‘biographical’ self), we propose that transitory SN desegregation and disintegration accounts for ketamine’s ‘*dissociative*’ state, while transitory DMN desegregation and disintegration accounts for ketamine’s ‘*psychedelic*’ state. In psychedelic-assisted psychotherapy, a relaxation of the highest-level beliefs with psychotherapeutic support may allow a revision of pathological self-representation models, for which neuroplasticity plays a permissive role. Our account provides a multi-level rationale for using the psychedelic properties of ketamine to increase its long-term benefits.

## Introduction

Depression affects around 300 million people worldwide and it is currently the leading cause of years lived with disability (Malhi and Mann, 2018). A third of these patients do not improve with existing therapies and develop a condition known as treatment-resistant depression (TRD) (Gaynes et al., 2009, 2020; Nemeroff, 2007). Until recently, all treatments for depression took at least 1–6 weeks until symptomatic improvement occurs (Fournier et al., 2010; Taylor et al., 2006). However, in the last 20 years, two parallel lines of research have shown unprecedented promise by showing fast therapeutic benefits. These relate to the N-methyl-d-aspartate (NMDA)-antagonist ketamine, that has proven to exert antidepressant as well as anti-suicidal effects within hours after administration (Berman et al., 2000; Zarate et al., 2006), and the 5HT2A-receptor agonist psilocybin, a serotonergic (or classic) psychedelic, that has been studied particularly for major (Davis et al., 2021) and TRD (Carhart-Harris et al., 2016, 2021), showing promising, albeit preliminary, results.

Psychedelics (from the Greek *psyche* (mind) and *delius* (to manifest)) are substances that induce transient states of profoundly altered perception, thought and emotion, and have been used for thousands of years in ceremonial contexts in multiple cultures (Carod-Artal, 2015; Grinspoon and Bakalar, 2018; Sessa, 2005, 2007). Their effects are particularly dependent on the context in which they are administered (Johnson, Richards and Griffiths 2008; Nichols and Walter, 2021) – when administered in a safe context, a common acute effect is a disruption of ego-boundaries, which results in a blurring of the distinction between self-representation and object-representation, known as ‘ego dissolution’ (Anderson and Rawnsley, 1954; Cohen and Eisner, 1959; Denber, 2006; Eisner and Cohen, 1958; Klee, 1963; Lewis and Sloane, 1958; Millière, 2017; Nour et al., 2016). Clinical trials have demonstrated the effectiveness and safety of one or two administrations of psilocybin with psychological support in patients with depression in a revolutionary model that does not require prolonged daily administration of medication (Carhart-Harris et al., 2016; Davis et al., 2021). Pharmacologically, there is strong evidence that activation of serotonergic 5HT2A receptors is the core initial mechanism that mediates the profound and variable effects of all classic psychedelics (De Gregorio et al., 2018; Halberstadt et al., 2020; Kim et al., 2020; Kraehenmann et al., 2017; Vollenweider et al., 1998; Winter et al., 2007). The stimulation of 5HT2A receptors causes a *glutamate surge*, primarily in layer V pyramidal neurons expressing 5-HT2A receptors, and a sustained increase in neuroplasticity (De Gregorio et al., 2018; Lambe and Aghajanian, 2005; Ly et al., 2018; Muschamp et al., 2004; Scruggs et al., 2003). Results from neuroimaging studies have shown that psilocybin acutely increases signal diversity in the brain (Schartner et al., 2017), reduces low-frequency oscillations (Muthukumaraswamy et al., 2013) and modifies activity in major large-scale networks, including the salience network (SN), involved in ‘bodily’ self-experience (also known as *minimal* self), and the default-mode network (DMN), involved in ‘narrative’ self-experience (also known as *biographical* self) (Carhart-Harris et al., 2012; Lebedev et al., 2015; Letheby and Gerrans, 2017; Preller et al., 2020). It has also been demonstrated that psychedelic-induced experiences of ego dissolution are associated with long-lasting benefits and a greater reduction in depressive symptoms (Barrett and Griffiths 2018; Davis et al., 2020; MacLean et al., 2011; Roseman et al., 2018, 2019). In 2019, Carhart-Harris and Friston (2019) proposed an elegant unified computational model of serotonergic psychedelics’ effects in the brain. According to this model, which is based on hierarchical predictive processing (HPP) and the Bayesian brain hypothesis, classic psychedelics relax the precision weighting of high-level priors and liberate bottom-up information flow. Later, Letheby argued that long-lasting therapeutic benefits of psychedelic-assisted psychotherapy (PAP) for depression and other clinical conditions specifically occur when the relaxed and revised priors concern models of self-representation (Letheby, 2021). PAP is considered by many as a paradigm shift in psychiatric research and treatment (Nichols et al., 2017; Schenberg, 2018), and has been widely demonstrated as a safe and well-tolerated intervention (Aday et al., 2020; Rucker et al., 2022; Schlag et al., 2022). Nonetheless, no serotonergic psychedelic has been formally approved for any clinical indication and, thus, as controlled substances, its use is still restricted to the context of clinical trials.

Ketamine was first synthesized in 1962 by the Parke Davis’ organic chemist Calvin Stevens (Kolp et al., 2014). In its first human trials, clinicians found that at high doses, ketamine caused unconsciousness but at lower doses it produced peculiar psychoactive effects, with subjects describing being ‘spaced out’ and ‘floating’ (Domino et al., 1965, 1984; Haas and Harper, 1992). Importantly, this occurred in the middle of the 1960s, when classic psychedelics had started being used in poor controlled settings by the general population (Wolfson, 2014). Parke-Davis wanted to avoid characterizing the drug as psychedelic, and the term *dissociative anaesthetic* was suggested to describe the way it seemed to ‘separate the mind from the body’ (Domino, 2012). Interestingly, some researchers in the 1970s–1980s acknowledged similarities between some of ketamine’s subjective psychoactive effects and the effects of classic psychedelics – and the potential benefits of ketamine’s subjective experience for the treatment of depression and addiction, with promising results (Fontana and Loschi, 1974; Khorramzadeh and Lotfy, 1973; Krupitsky and Grinenko, 1997).

At the turn of the century, a group of researchers began to study the administration of ketamine, in a subanaesthetic dose, for the treatment of TRD, showing high efficacy in the reduction of depressive symptoms (Berman et al., 2000; Fava et al., 2018; Murrough et al., 2013; Price et al., 2009; Zarate et al., 2006). These studies used intravenous ketamine and found effects within hours following a single administration (Price et al., 2009; Fava et al., 2018; Murrough et al., 2013). When ketamine administration is repeated in the following weeks, efficacy can reach 70% (Bahji et al., 2021; Coyle and Laws, 2015; Fond et al., 2014; Kishimoto et al., 2016; McInnes et al., 2022). Multiple randomized placebo-controlled trials have shown that the intravenous administration of ketamine at the dose of 0.5 mg/kg has rapid, robust, albeit relatively short-lived antidepressant effects in patients with depression (Berman et al., 2000; Fava et al., 2018; Murrough et al., 2013; Price et al., 2009; Zarate et al., 2006). A meta-analysis of nine randomized controlled trials (RCTs) found that a ketamine infusion produces antidepressant effects that start 40 min after administration, attains a peak after 24 h and loses superiority over placebo around 10–12 days afterwards (Kishimoto et al., 2016). A ketamine enantiomer – esketamine – with intranasal administration was recently approved by the Food and Drug Administration and European Medicines Agency for TRD after several industry-sponsored trials with favourable results, although a recently published meta-analysis found that intravenous ketamine appears to be superior to intranasal esketamine for the treatment of depression (Bahji et al., 2021). In parallel, several studies suggest a possible beneficial effect of adding psychological therapy alongside ketamine treatment for depression and addiction. Wilkinson and colleagues performed a RCT of ketamine for TRD comparing a group which received ketamine and cognitive behavioural therapy (CBT) with a group that received ketamine and treatment as usual and found greater sustained improvement in the CBT group (Wilkinson et al., 2021). In individuals with cocaine dependence, a single ketamine infusion plus behaviour modification promoted abstinence and reduced craving (Dakwar et al., 2014, 2018). A recently published trial of ketamine combined with mindfulness-based relapse prevention for alcohol use disorder demonstrated that treatment with three infusions of ketamine was associated with more days of abstinence from alcohol at 6-month follow-up (Grabski et al., 2022). After reviewing these and other studies, Muscat et al. (2022) have recently argued for the potential benefits of integrating ketamine protocols with psychotherapy.

Interestingly, it has been recently reported that positive-valenced ego dissolution experiences during ketamine administration are associated with greater antidepressant response (Aust et al., 2019; Sumner et al., 2021). Also, in separate studies, it was demonstrated that ketamine produces neurobiological effects which are remarkably similar to the effects of serotonergic psychedelics – reduced activity in the SN and in the DMN (Bonhomme et al., 2016; Mueller et al., 2018; Zacharias et al., 2020), reduced low-frequency oscillations (Vlisides et al., 2018; Zacharias et al., 2020) and increased signal diversity (Schartner et al., 2017). With the goal of achieving sustained long-lasting results, several clinical centres have started offering PAP using ketamine – a model known as ketamine-assisted psychotherapy (KAP). However, despite the extensive amount of evidence regarding its neurobiological mechanisms and the recently produced evidence regarding the relevance of its acute psychological effects, no unified account has been proposed for ketamine. A computational framework which defines what is being represented across different levels can help to sketch a unified picture (from molecules to subjective experience) for ketamine, as has been proposed for serotonergic psychedelics. Here, we propose a multi-level model for ketamine’s effects and therapeutic potential which is based on the existent research and generates testable predictions. We start by reviewing the neurobiological and psychological findings for ketamine and, afterwards, we offer a computational account of ketamine’s action in the brain which tries to explain its dissociative and psychedelic properties, as well as its antidepressant effects. After discussing its implications and limitations, we suggest how it might be tested and, if the model holds, how ketamine’s benefits could become more sustained.

## Narrative review of main findings in ketamine studies

### Molecular and cellular levels

In addition to its principal mechanism of action as a non-competitive antagonist of NMDA receptors (binding at the PCP site), ketamine also exhibits action at muscarinic, opioid and adrenergic receptors; also blocking potassium/sodium hyperpolarization-activated cyclic nucleotide-gated (HCN1) channels as well as the serotonin and norepinephrine transporters (Bergman, 1999; Chen et al., 2009; Muthukumaraswamy et al., 2015; Stahl, 2013). Strong evidence suggests that both its antidepressant and its dissociative anaesthetic properties are mediated by the NMDA antagonism (Aleksandrova and Phillips, 2021; Autry et al., 2011; Duman et al., 2012; Kadriu et al., 2021). However, as other NMDA antagonists such as memantine do not have the same effects, activity at other receptors may also play a role. Interestingly, it has been shown that ketamine is a likely agonist of 5HT2A receptors, with binding affinities close to those at NMDA receptors (Kim et al., 2000; Lin et al., 2018).

There are two main hypotheses regarding the consequence of ketamine’s NDMA antagonism and these hypotheses are not considered mutually exclusive (Aleksandrova and Phillips, 2021). According to the ‘disinhibition’ view, ketamine blockade of NMDA receptors on GABAergic interneurons causes a consequent reduction of inhibitory control over pyramidal neurons (Abdallah et al., 2020; Gerhard et al., 2020; Homayoun and Moghaddam, 2007; Maeng et al., 2008). This view is based on the initial findings that the administration of ketamine in rats increases glutamate release in the medial prefrontal cortex (PFC) concomitant with an increase in the firing rate of pyramidal neurons in this brain area (López-Gil et al., 2007; Moghaddam et al., 1997). AMPA receptor antagonists or mGluR2/mGluR3 agonists block the behavioural effects of NMDA antagonists in rats, suggesting that (at least a subset) of ketamine’s effects are due to increased extracellular glutamate levels – which has been named ‘a glutamate surge’ (Moghaddam et al., 1997; Moghaddam and Adams, 1998; Razoux et al., 2006). Evidence that an increase in extracellular glutamate after ketamine causes an increase in AMPA activation relative to NMDA activation also supports this view (Maeng et al., 2008). There is also an alternative view, the ‘direct inhibition’ view, which proposes that ketamine’s antagonism of NMDARs directly on pyramidal neurons at rest blocks tonic NMDAR activation by ambient or spontaneously released glutamate, which, in turn, reduces the suppression of protein synthesis and activates downstream synaptogenic cascades (Kavalali and Monteggia, 2012; Nosyreva et al., 2014). There are also contradictory findings, as some studies have proposed that increases in glutamate in the PFC may be used as markers of clinical response to ketamine, while others have recently shown that ketamine decreases glutamate levels in the cortex (Abdallah et al., 2014; Lazarevic et al., 2021; Zanos et al., 2016). Importantly, ketamine increases evoked excitatory postsynaptic potentials/currents in layer V pyramidal neurons, just like psilocybin (Aleksandrova and Phillips, 2021).

There is strong evidence that ketamine induces synaptic, structural and functional changes in pyramidal neurons (Aleksandrova and Phillips, 2021). In animal models, ketamine increases brain-derived neurotrophic factor (BDNF) levels in the cortex in a dose-dependent fashion and loses its antidepressant effects in BDNF knock-out mice (Kim et al., 2021; Yang et al., 2013; Zhou et al., 2014). There is also evidence that ketamine reduces the suppression of eukaryotic elongation factor 2-mediated protein synthesis and engages similar downstream synaptogenic cascades (Autry et al., 2011; Kavalali and Monteggia, 2012; Kim et al., 2021; Zhou et al., 2014). After BDNF being released, it binds to TrKb receptors and increases mTOR signalling via MEK-ERK and Akt (Castrén and Monteggia, 2021; Girgenti et al., 2017; Li et al., 2010). It has also been demonstrated that mTOR activity is increased after ketamine and that mTOR inhibition eliminates its synaptogenic and antidepressant effects (Li et al., 2010; Zhou et al., 2014). In humans, one study found that subjects who respond to ketamine treatment for depression show higher levels of BDNF (24 h after administration) than non-responders and that there was a negative correlation between BDNF levels and Montgomery-Asberg Depression Rating Scale (MADRS) score (Haile et al., 2014). Contrary to what was expected, in a recent report, blocking mTOR signalling did not preclude ketamine’s antidepressant effects in humans but extended its antidepressant effects (Abdallah et al., 2020).

Ketamine-induced activation of AMPA receptors is associated with the increase in BDNF and mTOR signalling causes an increase in neuroplasticity-related gene expression and protein synthesis (Aleksandrova and Phillips, 2021; Kavalali and Monteggia, 2012; Kim et al., 2021; Li et al., 2010; Nosyreva et al., 2014). In rodents, ketamine increases dendritic complexity, spine density and synaptic strength for 2 weeks (Moda-Sava et al., 2019; Phoumthipphavong et al., 2016; Wu et al., 2021). Cavalleri et al. (2017) have also shown that it may also enhance structural plasticity in dopaminergic neurons. In animal models of depression, ketamine reverts behavioural correlates of depression 30 min after the injection, an effect that lasts for around 1 week (Yang et al., 2013). Also, ketamine (0.5 mg/kg) is able to rescue long-term potentiation deficits in a rat model of stress susceptibility, with consequent synaptic strengthening on the day after (Aleksandrova et al., 2020). In humans, Sumner et al. (2020) have shown that ketamine increases long-term potentiation (LTP) based neural plasticity 24 h after administration. Thus, ketamine’s effects in neuroplasticity are very similar to the effects of psilocybin and other serotonergic psychedelics, although there may be differences in the duration of these effects (Aleksandrova and Phillips, 2021; Kadriu et al., 2021).

### Circuit and network levels

An important result was reported by Yang et al. (2018), who have demonstrated that ketamine-induced blocking of NMDAR-dependent bursting activity of neurons in the lateral habenula (LHb) disinhibits downstream monoaminergic reward centres. The LHb is a basal ganglia nucleus known as the ‘anti-reward’ centre; thus, it has been argued that this effect mediates the almost immediate antidepressant effect of ketamine (Pulcu et al., 2021). LHb neurons show an increase in burst activity and theta-band synchronization in rodents with depressive phenotypes, which is reversed by ketamine (Yang et al., 2018). While burst-evoking photostimulation of LHb drives behavioural despair and anhedonia, Yang et al. have demonstrated that LHb bursting requires both NMDARs and low-voltage-sensitive T-type calcium channels (T-VSCCs) and that local blockade of NMDA receptors or T-VSCC’s in the LHb is sufficient to induce rapid antidepressant effects. Importantly, this effect disappears when the substance is cleared from the organism (half-life = 2.5–3 h (Mathew and Zarate, 2016; Sinner and Graf, 2008).

The first study analysing resting-state functional connectivity with ketamine demonstrated that, in healthy subjects, ketamine reduces activity in the DMN 24 h after the infusion (Scheidegger et al., 2012). Bonhomme et al. (2016) compared resting-state networks connectivity during stepwise infusions of ketamine in healthy volunteers and found reduced intra-DMN and intra-SN connectivity and a suppression of the anti-correlation between the DMN and other networks 24 h after the infusion (Bonhomme et al., 2016). More recently, other studies have provided clear evidence that ketamine administration contributes to acute disintegration of DMN functional connectivity (Mueller et al., 2018; Zacharias et al., 2020). An increase in the connectivity between the CEN and resting-state networks (DMN and SN) in healthy individuals was reported by Mueller et al (2018). Zacharias et al. (2020) performed a multimodal study of functional magnetic resonance imaging (fMRI) and electroencephalogram (EEG) during ketamine administration and found, in healthy volunteers, decreased functional connectivity in the medial PFC and increased functional connectivity in the intraparietal cortex, accompanied by an EEG shift to delta, gamma and theta waves (reduction in alpha power). In this study, frontal connectivity was negatively correlated with EEG gamma and theta activity, while a positive correlation was found between parietal connectivity and EEG delta power. These studies suggest that ketamine decreases within-network functional connectivity and increases between-network functional connectivity, similar to psilocybin.

In depressed individuals, Abdallah and colleagues found that those who responded to ketamine showed increased global signal regression (GBCr) in the lateral PFC, caudate and insula 24 h after the infusion (Abdallah et al., 2016) and that GBCr in the PFC positively predicted depression improvement (Abdallah et al., 2018). However, Kraus et al. (2020) failed to replicate this finding 48 h after the dosing session in a different sample. Interestingly, the latter group recently reported that ketamine increased frontostriatal connectivity in major depressive disorder (MDD) patients towards levels observed in a control group of healthy volunteers while shifting the connectivity profile of healthy individuals towards a state which was similar to TRD patients under placebo (Mkrtchian et al., 2020). A brain region which has important connections with striatal circuits and has been previously implicate in depression is the anterior cingulate cortex (ACC) and several neuroimaging studies have found differences in subgenual and peri-genual ACC activity after ketamine infusions, some of them correlating with antidepressant effects (Alexander et al., 2021).

In a MEG study, Muthukumaraswamy et al. found that sub-anaesthetic doses of ketamine were associated not only with decreases in occipital, parietal and anterior cingulate alpha power oscillations, but also in NMDA and AMPA-mediated fronto-parietal connectivity (Muthukumaraswamy et al., 2015). Remarkably, they also found a decrease in synaptic gain in the parietal cortex which was correlated with subjects’ ratings of a blissful state, a finding which was also present with psilocybin in another study by the same group (Muthukumaraswamy et al., 2013). A very relevant study assessed differences in MEG and subjective experience between ketamine, psilocybin and LSD (Schartner et al., 2017). All substances produced higher spontaneous signal diversity, captured by the Lempel-Ziv complexity score (which quantifies the number of distinct patterns present in data – and has been previously shown to be reduced in propofol-induced anaesthesia and deep sleep). In fact, in the correlation between subjective self-reports designed to capture the full breadth of psychedelic states and MEG-extracted measures, ketamine achieved the strongest correlation between subjective ratings of psychedelic experience and spontaneous signal diversity (even higher than psilocybin and LSD) (Schartner et al., 2017). The authors mentioned that the shared phenomenological and electrophysiological effects across these different subjects may be mediated by the interactions between 5HT2A and NMDA receptors or, alternatively, and according to more recent perspectives, that 5HT2A agonism and NMDA antagonism may have similar effects on the activity of neuronal populations in the cortex. A complementary and interesting report by Li and Mashour, who administered low (psychedelic) and high (anaesthetic) doses of ketamine in a MEG scanner (Li and Mashour, 2019). The authors found that the subanaesthetic dose of ketamine was associated with an elevated complexity level relative to baseline, while the brain activity following an anaesthetic dose of ketamine is characterized by alternating low and high complexity levels until stabilizing at a high level comparable to that during baseline (Li and Mashour, 2019). In another study, a machine learning algorithm was able to differentiate between ketamine and serotonergic psychedelics using magnetoencephalography (MEG) data (Pallavicini et al., 2019). The authors found a pattern of occipital, parietal and frontal decreases in the low alpha and theta bands that were specific to lysergic acid diethylamide (LSD) and psilocybin, as well as decreases in the low beta band common to the three drugs. More recently, the same dataset was used to compared ketamine, psilocybin, LSD and the non-psychedelic anticonvulsant tiagabine using separate measures for directed functional connectivity measures and undirected functional connectivity (Barnett et al., 2020). A general decrease in directed functional connectivity was found for ketamine and serotonergic psychedelics but not for tiagabine, as measured by Granger causality, throughout the brain. The authors concluded that the psychedelic state, which can be reached through the ketamine, psilocybin or LSD, involves a common breakdown in patterns of functional organization or information flow in the brain (Barnett et al., 2020). They state that a disintegration of communication between and within brain regions, which, in turn, implies a loosening of dynamical constraints on brain activity in psychedelic states, may correspond to an enlarged repertoire of dynamical states, in line with *the entropic brain hypothesis* (Carhart-Harris et al., 2014), which links increased dynamical diversity to the characteristic subjective effects of psychedelics including unconstrained cognition, perception and ego dissolution.

### Psychological level

To explore the acute subjective experience of psychedelics, Vollenweider and Kometer (2010) administered different dosages of ketamine (6 µg per kg per min or 1.2 per kg per min intravenously) and psilocybin (115–125; 215–270 and 315 µg per kg orally) to healthy individuals and applied the five dimensions version of the Altered States of Consciousness Scale (5D-ASC) (Vollenweider and Kometer, 2010). The authors concluded that these compounds produce a set of overlapping dose-dependent psychological experiences, including a pleasurable loss of ego boundaries and feelings of oneness or a more psychotic-like ego dissolution that involves fear and paranoid ideation (Vollenweider and Kometer, 2010). However, Studerus et al. later found some differences using the 11 dimensions version of the ASC (Studerus et al., 2010). Ketamine frequently produced more intense dissociative-like bodily experience, captured by the ‘disembodiment’ factor of the 11D-ASC, while serotonergic psychedelics cause greater alterations in sensory experiences in the visual domain. The ketamine subjective state was also associated with lower scores in the ‘blissful’ factor and higher scores in ‘impaired control and cognition’. Importantly, ketamine facilitated unitive and spiritual experiences to the same degree as psilocybin (Studerus et al., 2010).

Regarding the clinical use of ketamine, the vast majority of studies followed an exclusively pharmacological model, in which subjective effects during administration of the substance are seen as side effects. Nevertheless, some of these studies have used psychometric instruments to analyse the acute psychological experience during ketamine administration and correlated these scores with change in depression scales. Three types of scales have been used: the Clinician-Administered Dissociative States Scale (CADSS), the Brief Psychotic Rating Scale (BPRS) and the 5D-ASC. Mathai et al. reviewed studies using single ketamine infusions and concluded that two (Luckenbaugh et al., 2014; Phillips et al., 2019) of five studies (Fava et al., 2018; Lapidus et al., 2014; Valentine et al., 2011) using CADSS found a significant negative correlation between dissociative experiences and depression scores. Regarding psychotic symptoms, only one of the six BPRS studies found a significant (negative) correlation between BPRS score and depression measures (Sos et al., 2013). Mathai only included one study using the 5D-ASC, a scale which captures a broad range of subjective experiences, and that study did not find a significant correlation, but the sample size was very small to allow any conclusions to be extracted (Vidal et al., 2018). More recently, using the 5D-ASC and repeated ketamine infusions, Aust and colleagues have demonstrated that dread of ego dissolution (anxiety related) experiences induced by ketamine were higher in non-responders, suggesting that the quality of the subjective experience is important for its therapeutic effects (Aust et al., 2019). Also, it has just been published a study (Sumner et al., 2021) which found that greater antidepressant response with ketamine was correlated with three ‘oceanic boundlessness’ sub-factors of the 11D-ASC scale: experience of unity, spirituality and insight. Finally, it had also been demonstrated that ketamine-induced mystical experiences are associated with improvements in cocaine-use disorder (Dakwar et al., 2014, 2018) and alcohol-use disorder (Rothberg et al., 2021).

In 2017, van Schalkwyk and colleagues complemented a quantitative analysis of the CADSS scale after ketamine infusions with qualitative interviews. Qualitative analysis of patient narratives uncovered important aspects of the subjective experience that were not captured by the CADSS, including sense of peace and disinhibition (van Schalkwyk et al., 2018). The abovementioned study by Sumner et al. (2021) complemented their quantitative assessment of ketamine experiences with the ASC scale with a qualitative interview. The first qualitative interview revealed that all participants experienced perceptual changes and additional themes included loss of control and emotional and mood changes. The final interview showed evidence of a psychedelic afterglow, and changes to perspective on life, people and problems, as well as changes to how participants felt about their depression and treatments.

Results of qualitative interviews obtained during a RCT of KAP for alcohol dependence have also been published (Mollaahmetoglu et al., 2021). Six key themes were identified: (1) multifaceted motivations to participate in the trial; (2) the influence of ‘set’ and ‘setting’ in the acute subjective experience; (3) the inherent contradictions of the ketamine experience; (4) rapidly fluctuating and changing experiences; (5) meaningful, spiritual and mystical experiences and (6) transformational effects of the infusions and the trial. Regarding the set as an influential component in the ketamine experiences, the participants’ expectations of the infusions, their prior mindset and spiritual beliefs seemed to have an impact on the acute experiences. Setting also seemed to play a key role in the experience with ketamine. The experience with ketamine also carries some contradictions, with participants describing experiences that were both highly positive and negative, comparing it to a ‘rollercoaster drive’. Among the rapidly fluctuating and changing experiences were perceptual distortions with dimensional and spatial distortions, changes in the perception of colour and texture and voices sounding further or closer than they were. Nearly all the participants described feelings of dissociation or detachment from their environment, their physical body or their sense of self. Some of them also reported phenomena of ego dissolution, with a diminishing sense of self-importance and of the self as a separate entity and feelings of unity with the rest of the universe, that were connected to the acquisition of deep and meaningful insights and new perspectives on their lives. Furthermore, some participants reported experiences of transcendence of time. Finally, the experience with ketamine was described by many of the participants as potentially transformative, both of their perspective in life and their relationship with alcohol and its dependence. If we follow the most accepted definition of psychedelics – substances which cause transient but profound changes in perception, thought and emotion that are not experienced otherwise except in dreams and at times of religious exaltation (Nichols, 2016) – then the studies of psychological effects of ketamine provide clear-cut evidence that, at least in some dosages and contexts, ketamine is a substance with psychedelic properties. What is lacking in a unified account which integrates all these separate – and at times seemingly contradictory – findings.

## Discussion

We will proceed from low-level molecular to network-level effects, followed by psychological aspects, discussing the similarities and differences between ketamine and serotonergic psychedelics. In this discussion, we will mainly focus on psilocybin, the most studied classic psychedelic for the treatment of depression. Then, we will leverage on the unified computational accounts of classic psychedelics to propose a model which integrates the short- and long-term neurobiological and psychological effects of ketamine in the brain.

### Ketamine acutely modulates reward circuits and sub-acutely increases neuroplasticity

An important property of both classic psychedelics and ketamine is their capacity to induce changes in neuroplasticity (Kadriu et al., 2021). In fact, Aleksandrova and Philips (2021) have recently proposed that psilocybin and ketamine have a common downstream mechanism of action: both inducing a burst of glutamate release and sustained AMPA activation in excitatory pyramidal neurons. According to these authors, these effects potentiate BDNF and mTOR signalling, upregulating the expression of genes related to neuroplasticity and protein synthesis of synaptic components, triggering a fast amplification mechanism that drives local synaptogenesis. However, it seems that this effect of ketamine is more transient than for classic psychedelics and disappears after 8–10 days after a single infusion, a parallel evolution to the resurgence of depressive symptoms after a single dose of ketamine. This is why multiple infusions of ketamine are typically used in clinical practice and most studies using psilocybin have either tested one or two administrations of the drug.

The study by Yang et al. (2018), which has demonstrated the influence of ketamine on the LHb, has revealed a mechanism which can explain ketamine’s almost immediate antidepressant properties. More recently, Pulcu et al. (2021) provided an elegant translational account on the anti-anhedonic effect of ketamine in the LHb. Several studies have highlighted a possible function for the LHb as a relay station orchestrating signals from both dopaminergic and serotonergic regions that regulate affective response during reward-guided interactions with the physical environment. In fact, the LHb has been implicated in coding of negative emotions (Matsumoto and Hikosaka, 2007; Shabel et al., 2012) and is aberrantly hyperactive in individuals with depression (Li et al., 2011, 2013; Morris et al., 1999; Shumake and Gonzalez-Lima, 2003). Most LHb neurons are glutamatergic but they inhibit the brain’s reward centres through a relay in GABAergic interneurons in the dopaminergic ventral tegmental area or the serotonergic dorsal raphe nucleus (Jhou et al., 2009; Tian and Uchida, 2015; Zhou et al., 2017). The ACC also seems to be a key locus of ketamine’s rapid antidepressant action (Alexander et al., 2021) and it projects to the LHb (Chiba et al., 2001). Although the modulation of reward circuits is important to explain the immediate antidepressant effects of ketamine (sometimes detected in less than an hour after the dosing session), the maintenance of the effect in the following days needs to be explained by other mechanisms. An important aspect is that the NMDA antagonism at the LHb, which seems to be specific for ketamine and absent in serotonergic psychedelics, may explain its higher reinforcing effect and risk of addiction, particularly when used in recreational contexts without medical supervision (Liu et al., 2016; Morgan and Curran, 2012).

In an influential manuscript published in 2010, Vollenweider and Kometer argued that there are two opposite views on the relationship between psychedelics (ketamine included) and neuroplasticity. In one of these views, the increase in neuroplasticity is the central mechanism for (short term and long term) benefits of psychedelics by opening a period of increased adaptation in which it is easier to modify pathological cognitions or behaviours, particularly when combined with psychotherapy. In this framework, the psychedelic subjective experience is an epiphenomenon viewed as a side effect. The opposite view sees the psychedelic experience itself as the catalyst for neuroplastic changes. According to this account, in the same way as meditation and other practices can increase neuroplasticity (Davidson and Lutz, 2008; Tolahunase et al., 2018), the psychedelic state is the central catalyst of the process of change, which is then implemented, in part, by increases in BDNF and other neuroplastic factors. The main problem for the first hypothesis is the empirical evidence that the most robust predictor of clinical response to PAP is not the dose or any molecular correlate of neuroplasticity but psychometric scores in questionnaires accessing subjective experiences (such as mystical experiences, ego dissolution or emotional breakthrough) (Barrett and Griffiths, 2018; Davis et al., 2019, 2020; Murphy et al., 2022; Roseman et al., 2018; van Oorsouw et al., 2022). A systematic review of clinical and biological factors which could predict the response to psychedelics in clinical populations, Romeo et al. (2021) found that the subjective intensity of the psychedelic experience is the main predictive factor of response across all diagnosis. Subjective experiences with mystical-like qualities or emotional breakthroughs are associated with a reduction in depressive symptoms and increase in quality of life measures, while subjective experiences lacking this components are associated with poor therapeutic outcomes (Murphy et al., 2022; Romeo et al., 2021; Roseman et al., 2018). Moreover, the strength of the therapeutic relationship predicts the quality of the acute subjective psychedelic experience and the final outcomes of psilocybin-assisted therapy for depression (Murphy et al., 2022). This evidence is very hard to reconcile with the neuroplastic hypothesis – as molecular and cellular-level stimulation of neuroplasticity should be dependent on dose, not therapeutic relationship – and several authors have suggested that although these molecular mechanisms surely make a relevant contribution, they are not sufficient for long-lasting change (Yaden and Griffiths, 2021). Later in this article, we will propose a conciliatory view between these positions after the presentation of unified accounts. Regarding ketamine, if we take in consideration studies that used psychometric scales capturing ego dissolution or mystical experiences (ASC scale or Mystical Experience Questionnaire (MEQ)), similar evidence for an experience-dependent component is clearly mounting up (Aust et al., 2019; Dakwar et al., 2014, 2018; Sumner et al., 2021). We propose that ketamine’s immediate blocking of LHb bursting activity and modulation of ACC circuits are responsible for its immediate antidepressant effects, while the sub-acute (1 to 7–10 days) increase in neuroplasticity allows for the antidepressant effects to be maintained for a period of 1–2 weeks. However, as reviewed above, this molecule causes other dramatic acute effects at circuit and network levels in the brain, as well as in subjective experience, which allow us to draw a richer and more integrated picture.

### Ketamine reduces low-frequency oscillations, disrupts activity in large-scale functional networks and increases signal diversity

It is important to note that although the initial receptor in which classic psychedelics act to exert their effects is serotonergic, most of the downstream changes are caused by a glutamatergic mechanism (Carhart-Harris and Friston, 2019). By binding to 5HT2A receptors in layer V pyramidal neurons which are dispersed through the cerebral cortex, psilocybin modifies glutamate neurotransmission and desynchronizes its depolarization profile (Vollenweider et al., 1998). The neurophysiological correlate of this effect is the modification in oscillatory activity in alpha-band, detected in EEG and MEG, particularly in the PCC, a core node of the DMN (Muthukumaraswamy et al., 2013). Specifically, psilocybin reduces alpha power, increases gamma power and causes an overall brain dynamic characterized by increased between-network global functional connectivity, expanded signal diversity, and a larger repertoire of structured neurophysiological activation patterns (Muthukumaraswamy et al., 2013; Schartner et al., 2017; Swanson, 2018). The circuit-level effects of this modification are the disintegration and desegregation of resting-state functional networks, increasing communication between functionally distinct areas and decreasing communication between functionally similar areas – a pattern of compromised modular but enhanced global connectivity which has been subsumed under *the entropic brain* hypothesis (Carhart-Harris et al., 2014; Carhart-Harris, 2018). The most commonly cited neuroimaging correlate of this acute effect is the modification in functional connectivity in the DMN (Lebedev et al., 2015; Smigielski et al., 2019). Curiously, Lebedev et al. (2015) found that higher loadings in a self-report ‘ego dissolution’ factor were associated with a ‘disintegration’ of the SN. To reconcile these findings implicating the SN with previous reports implicating the DMN, the authors argue that the DMN is more related to the narrative self while the dynamics of the SN (which includes nodes such as the ACC, anterior insula and amygdala) may promote other aspects of self-representation falling under the construct of ‘minimal’ or ‘embodied’ self. After treatment, in the first trial of psilocybin for TRD, the authors found increased functional connectivity within the DMN after treatment (Carhart-Harris et al., 2017). Importantly, these last findings suggest that psilocybin decreases DMN integrity acutely and increases DMN integrity (or normalizes it) post-acutely, accompanied by improvements in mood. In the trial of psilocybin versus escitalopram for MDD, analysis of fMRI data showed that response to psilocybin correlated with decreases in large-scale network modularity, suggesting that psilocybin’s antidepressant action may depend on a global increase in how different brain networks are integrated (Daws et al., 2022).

It is striking how similar findings have been obtained with ketamine: it reduces alpha power (Vlisides et al., 2018), increases gamma power (Gilbert and Zarate, 2020) and augments signal diversity (Schartner et al., 2017), similar to psilocybin. The increase in signal diversity induced by these compounds is particularly notable, since the measure which was used (Lempel-Ziv complexity) is an operationally useful one-dimensional scale for level of consciousness, with wakeful rest and rapid eye movement sleep (previously considered to be) at the top and coma and propofol-induced general anaesthesia at the bottom. The increases in Lempel-Ziv complexity induced low-dose ketamine, psilocybin and LSD represent the first observations of an increase in theoretically motivated measures of conscious level with respect to the baseline of wakeful rest (Schartner et al., 2017). Ketamine also causes desegregation and disintegration of DMN and SN in healthy volunteers (Bonhomme et al., 2016; Mueller et al., 2018; Zacharias et al., 2020) and post-treatment functional connectivity modifications in the SN and the DMN were reported in depressed subjects (Evans et al., 2018; McMillan et al., 2020). It has been proposed that narrative/autobiographical self-representation is implemented by the DMN (Carhart-Harris and Friston, 2010) and that the SN represents the ‘minimal’ or ‘embodied’ self (Lebedev et al., 2015; Letheby and Gerrans, 2017). According to the computational framework of predictive coding (which we will detail later), it has been demonstrated that the anterior insula, a core region of the SN, plays a key role as a comparator mechanism sensitive to interoceptive prediction error signals, as informing visceromotor control, and as underpinning conscious access to emotional states (Seth, 2013). Letheby and Gerrans have proposed that serotonergic psychedelics cause a disruption of the typical functioning of the SN which leads to changes to body boundaries, spatial self-location and personal relevance of emotional feelings (Lebedev et al., 2015; Letheby and Gerrans, 2017; Tagliazucchi et al., 2016) and a disruption of the DMN typical functioning which leads to a dissolution of the narrative self (personality, history, goals and ownership of thoughts) (Bouso et al., 2015; Carhart-Harris and Friston, 2010; Carhart-Harris et al., 2012; Palhano-Fontes et al., 2015; Speth et al., 2016; Tagliazucchi et al., 2016). Importantly, with classic psychedelics, SN disruption seems to occur before DMN disruption (Preller et al., 2020), a finding which is in line with the classical descriptions that the *bodily* effects of psychedelics are felt before the *mental* effects (Savage, 1955). Similarly, the neuroimaging findings of ketamine studies reviewed in this article suggest that it has the capacity to disrupt both the SN, which is involved in the experience of body ownership, and the DMN, which involved in the creation of an autobiographical self-concept (Bonhomme et al., 2016; Evans et al., 2018; Mueller et al., 2018; Zacharias et al., 2020). Thus, we propose that the so-called ketamine’s ‘dissociative’ experiences occur by disruption of SN and ketamine’s ‘psychedelic’ experiences occur by disruption of DMN.

### Ketamine may cause transitory ego dissolution and a subsequent revision of self-representation

Turning for the psychological experience in more detail, if we focus on the themes identified in psilocybin-assisted therapy for depression (Watts et al., 2017) and search for them in the patient accounts of KAP for alcohol dependence (Mollaahmetoglu et al., 2021), which is the controlled trial in which ketamine was embedded in a psychotherapeutic framework with more similarities to the PAP model, we are able to find some clear parallels. For example, in the first trial of psilocybin for depression, most patients reported how depression made them feel ‘trapped in their minds’, disconnected from themselves, their senses, others and the world and how psilocybin made them feel connected to their selves, senses, others, the world and to a spiritual principle (Watts et al., 2017). In the ketamine trial, several patients achieved ego dissolution and described experiences of connection with ‘all living beings, people, things, the world and the universe’ (Mollaahmetoglu et al., 2021). In the psilocybin trial, most patients described that during dosing sessions, they experienced intense emotions of different affective qualities (joy, fear, terror, compassion, love and bliss) – with some patients describing the experience as an emotional rollercoaster – and that after the sessions they felt that their emotional repertoire was expanded, with long-lasting openness to emotional experiences. In the ketamine study, an important theme was the inherent contradiction of the experience, with positive feelings of calmness, peace and relaxation and negative emotions such as fear or panic. Another important theme was how the intense experience was seen as transformational, providing epiphanies and enlightenment, and modifying perspectives about life and emotions. On a different level, in the psilocybin study, patients commented on the importance of the preparatory sessions, on how music enabled them to surrender and accept emotions, how they felt connected to their therapists and valued the psychotherapeutic integration sessions which helped them weave a story of what happened. Similar perspectives on treatment were given by subjects in the ketamine trial, with several patients referring how ketamine interacted positively with psychotherapy, that one possible transformational effect of ketamine was making them more willing to engage and more open-minded towards the psychotherapy sessions, and most attributed the effects of the trial to both, seeing them as mutually supportive. Nevertheless, there were some important differences which we will discuss later: the duration of the acute effects (~1 h for ketamine and ~4 h for psilocybin) and the ketamine induced dissociative/disembodiment state described as ‘floating’/’detachment’ that is not typically described with psilocybin intake.

Regarding quantitative research, mystical-like experiences have strong evidence for mediating psilocybin therapeutic outcomes in clinical populations including depression (Carhart-Harris et al., 2017; Davis et al., 2019; Murphy et al., 2022; Roseman et al., 2018; van Oorsouw et al., 2022), addiction (Bogenschutz et al., 2015; Johnson et al., 2014, 2017; O’Reilly and Funk, 1964) and existential anxiety (Griffiths et al., 2016; Kurland et al., 1972; Pahnke et al., 1969; Richards et al., 1977; Ross et al., 2016). In healthy populations of psychedelic users, mystical experiences are also associated with long-lasting benefits, including enhanced psychological well-being, life satisfaction and life meaning/purpose in addition to positive enduring changes in mood, attitudes and behaviour (Griffiths et al., 2006, 2008, 2011, 2018; Haijen et al., 2018; MacLean et al., 2011; Uthaug et al., 2019). Some authors have proposed that mystical experiences are the fundamental therapeutic mechanism leading to positive clinical outcomes, regarding psychedelics as one (effective and reliable) of many ways of inducing mystical experiences (others being intense meditation, ascetic practises, near death experiences or spontaneously occurring mystical experiences) (Richards, 2016). The dose-independent correlation between mystical-type experiences and lasting psychological benefits has strong implications – it suggests that the core therapeutic mechanism for lasting benefits in psychedelic therapy has an experiential component, not an exclusively pharmacological process which is independent from acute subjective experience. However, the term *mystical* is problematic because it may suggest to some readers that the experience has supernatural origins. An additional problem is the recently produced evidence that psychedelics may shift metaphysical beliefs towards a non-naturalistic world view (Timmermann et al., 2021).

It has recently been proposed that classic psychedelics exert their therapeutic effect not by the mystical experience per se but by disrupting and allowing subsequent revision of the mental representation of the self (Letheby, 2021). In this view, to which we subscribe, modifications in the sense of self are the hallmark of mystical-type experiences that is common to transcendental visions or feelings of cosmic consciousness and to more naturalistic experiences of ego dissolution (Nour et al., 2016), emotional breakthrough (Murphy et al., 2022; Roseman et al., 2019) and psychological insight (Davis et al., 2020; Peill et al., 2022), which have also been reported to predict positive outcomes. In fact, close inspection of the phenomenological accounts of patients who responded to psychedelic-assisted therapy (Belser et al., 2017; Nielson et al., 2018; Watts et al., 2017) provides clear evidence that the most relevant aspects of the subjective experience concern modifications in how they view themselves. The change from disconnection (between themselves and others) to connection and from avoidance of emotionally self-relevant material to acceptance are clear examples of how the core mechanism of psilocybin-assisted therapy is the modification in self-representation (Watts et al., 2017). Additional data supporting this hypothesis come from a qualitative study by Amada and colleagues, who investigated narrative reports of individuals who had psychedelic experiences (Amada et al., 2020). Themes included decentred introspection, greater access to self-knowledge, positive shifts in self-evaluation processes, greater psychological and behavioural autonomy, and enhanced connectedness with others and the world. This study offers further support for the explanation that changes to narrative self are a cornerstone of psychedelics’ therapeutic and transformative potential.

The psychometric evidence for an experience-dependent effect for ketamine is less strong than for classic psychedelics, as most studies have used questionnaires that assess pathological symptoms (dissociative and psychotic) which are just a small part of the wide range of ketamine’s potential subjective effects – and that do not seem to play an important role in mediating ketamine therapeutic outcomes. However, as research groups have started to use the broader ASC scale in clinical samples, it seems that more positive-valence ego-dissolution experiences and less negative-valence ego-dissolution experiences are associated with better therapeutic outcomes (Aust et al., 2019; Dakwar et al., 2014, 2018; Rothberg et al., 2021; Sumner et al., 2021). These findings suggest that, at least in some subjects and therapeutic contexts, the psychological mechanisms underlying the efficacy of ketamine in treating depression are similar to the ones at play under psilocybin. Thus, as these components are increasingly contemplated in recent studies, it seems clear that long-lasting benefits after ketamine may also have an experience-dependent component. Additional evidence supporting this claim comes from the fact that the therapeutic effects of other NMDA antagonists with less dissociative effects were only modest (Iadarola et al., 2015). A recent meta-analysis has also shown that esketamine, which has less dissociative effects, is less effective than racemic ketamine for TRD (Bahji et al., 2021).

The poor evidence for correlation between dissociative symptoms (which can be seen as a modification in bodily self-representation) and therapeutic outcomes found in the systematic review published in 2020 by Mathai et al. (2020) is in line with our proposal that psychedelic (which can be seen as modification in narrative self-representation) effects of ketamine increase long-lasting benefits, as scales assessing ego-dissolution and related constructs were only applied in a negligible amount of all clinical trials. In fact, research with qualitative interviews in patients after receiving ketamine strongly suggests that the CADSS and the BPRS do not capture fundamental aspects of subjective experience which patients considered to be therapeutic (Mollaahmetoglu et al., 2021). The themes identified by patients are in fact very similar to those identified in psilocybin trials, supporting the idea that, at least in some cases, ketamine may attain positive outcomes by the same psychological mechanism: disruption and revision of self-representation. These studies highlight the importance of considering the subjective quality of the transitory altered state of consciousness that occurs during ketamine administration in depressed individuals (Aust et al., 2019; Mathai et al., 2020). They also suggest that at least some of the acute psychological effects should not be seen as side effects (as others have suggested (Ballard and Zarate, 2020)) but as an integral part of the therapeutic process. Notably, it has long been demonstrated that music increases the quality of the ‘emergence phenomenon’ when ketamine is used as an anaesthetic (Kumar et al., 1992) and a recent trial where ketamine was administered without preparation and music support in depressed patients had to be interrupted (Gálvez et al., 2018). Thus, embedding the medicine in a treatment context with adequate previous preparation, comfortable and supportive therapist-guided dosing sessions and post-ketamine psychotherapeutic sessions to discuss the acute experience may increase therapeutic efficacy.

Based on the literature discussed in this manuscript, we propose that ketamine:

- May induce changes in bodily/minimal self-awareness, relatively independently from the context of administration, often reported as a ‘floating’ or detached sensation, partly captured by the CADSS scale and by the ‘disembodiment’ factor of the ASC, commonly interpreted as *dissociative effects*.
- May induce changes in ‘narrative’ or mental self-experience, more dependent on the context of administration, possibly captured by the MEQ/HMS or the oceanic boundlessness factor of the ASC scale, commonly interpreted as *psychedelic effects*.

We also propose that the changes in narrative self-experience – more than the changes in bodily self-experience – may mediate long-lasting psychological benefits, as long as they are properly integrated. Finally, we will try to unify the effects of ketamine in the brain with its psychological counterparts using a computational framework.

### Ketamine’s dissociative state is caused by a relaxation of ‘bodily’ self-representation priors encoded in SN nodes and ketamine’s psychedelic state is caused by a relaxation of ‘narrative’ self-representation priors encoded in DMN nodes

To bridge the gap between neurobiological and psychological levels of explanation, it is necessary to turn to the conceptual framework which is currently the most accepted unified theory for brain function: HPP. HPP is a computational framework that views the brain as an inference system that builds hierarchical models of the world and uses them to predict its future inputs (Friston, 2005, 2009; Friston et al., 2007; Friston and Kiebel, 2009). According to this theory, the brain implements generative models which are best-guess statistical approximations for the hidden causes of its input (either from outside the body or inside the body) based on Bayesian principles of belief/model updating (Friston, 2005; Rao and Ballard, 1999). Importantly, in HPP, each belief has an associated precision (the statistical equivalent of the inverse variance), which can be comprehended as *felt confidence*. Physiologically, precision is thought to be encoded by the sensitivity or postsynaptic gain of the neural populations that encode predictions or prediction errors. Lower levels of the hierarchy model concrete perceptual features in small spatiotemporal scales and higher levels of the hierarchy model increasingly abstract features in larger spatiotemporal scales. These models are updated according to error detection and correction: each level attempts to predict the activity of the level below and cognitive processing is driven by the imperative to minimize bottom-up prediction errors (reducing the brain’s ‘free energy’). The closer the interoceptive or exteroceptive input is to the higher-level prior/model, the lesser the magnitude of the ‘prediction’ error, the lesser our model update and the stronger our felt confidence in its contents. The neurally plausible theory for belief updating in the brain is called predictive coding and has been fundamental to explain a wide account of data, from perception to cognition (Fletcher and Frith, 2008; Zeller et al., 2015). According to predictive processing, the contents of our conscious experience are just a small subset of the contents of the high-level generative model assigned the highest probability by the brain, according to its multimodal input to date. Thus, phenomenal awareness can be seen as a ‘controlled hallucination’ – *controlled* because the models that inhabit our consciousness are constrained by the extensive amount of sensory input (Metzinger, 2003b; Seth, 2013, 2021). Crucially, conscious representations are *transparent*, meaning that they are not experienced as representations (Metzinger, 2003a, 2003b). However, in some conditions, they can become *opaque* (meaning that their representational nature becomes apparent): states as lucid dreams, intense meditation, breathwork and, with higher reliability, after the administration of substances with psychedelic properties (Letheby, 2021; Seth, 2021).

The most comprehensive account of the action of psychedelics in the brain was advanced by Carhart-Harris and Friston (2019). In brief, the ‘Relaxed Beliefs Under pSychedelics’ (REBUS) model proposes that by activating 5HT2A receptors in the cortex, classic psychedelics disrupt the DMN, where these abstract representations are encoded, modifying the precision of higher-level models and then allowing these priors to be revised (Carhart-Harris and Friston, 2019). Due to its molecular pharmacology and the distribution of the 5HT2A receptors in the brain – densely expressed in the visual cortex and in cortical areas which are part of the DMN (Beliveau et al., 2017; Watakabe et al., 2009) – serotonergic psychedelics start by causing unusual visual experiences and quickly progress to profound subjective experiences which may include the dissolution of the ego/self. As there is extensive evidence that DMN activity is associated with self-referential processing and autobiographical processes, which are a fundamental part of conscious experience, it follows that the disruption of this system will cause a modification of phenomenal consciousness. By modifying the neuronal synaptic gain, psilocybin and other classic psychedelics relax the precision weighting of high-level priors, and create a transitory state where these models are imbued with diminished confidence (Kanai et al., 2015). Lower confidence in the highest levels of the cortical hierarchy allows for bottom-up prediction errors to travel up in the hierarchy more freely, hence the full name of the model: REBUS *and the anarchic brain*. This is because in the ordinary state of consciousness, precise high-level beliefs have an important constraining influence on the lower levels of the hierarchy, inhibiting their expression. In a psychedelic state, lower-level prediction errors, either from the outside environment or from inside the brain (e.g. the limbic system), reach conscious awareness. The increased sensitivity of the higher cortical levels allow the upward flowing prediction errors to modify the encoded priors, enabling the revision or updating of neuronally encoded beliefs. The decrease in top-down and increase in bottom-up information flow is in line with the evidence for psychedelically induced reductions to brain-wide top-down signalling (Alamia et al., 2020), directed information flow (Barnett et al., 2020; Preller et al., 2019) and hierarchical information flow (Girn et al., 2020, 2021; Luppi et al., 2021; Parker Singleton et al., 2021). As the precision weighting of these high-level priors must first be relaxed, before they can be revised, a safe psychotherapeutic container is fundamental to such a potentially destabilizing process.

By extensively reviewing psychometric and phenomenological studies of psychedelic-assisted therapy, Letheby (2021) builds on the REBUS model and elegantly argues that the central driver of therapeutic change is the modification in the sense of self (Letheby, 2021). Computationally, this should correspond to a decrease in precision-weighted high-level priors, specifically priors that integrate stimuli across different modalities and timescales by attributing them to a single underlying entity, which is the Self. This argument is partially based on an influential manuscript by Letheby and Gerrans in which the authors integrate the notions of ‘binding’ (a phenomenon involving the integration of different types of information across the brain) with predictive processing and propose that the self-model represents a persisting object to unify, make sense of, and predict ongoing patterns of salient, egocentric and autobiographical experience – an account they name predictive self-binding theory (Letheby and Gerrans, 2017). The profound symmetry between object-representation and self-representation in the brain accounts for the fact that the highest levels in the cerebral hierarchy minimize upcoming prediction errors by modelling the existence of a single underlying entity, which is the Self (Letheby and Gerrans, 2017). Letheby argues that by reducing the brain’s confidence in its expectations about reality, including the Self, psychedelics decrease the influence of those beliefs on the contents of phenomenal awareness (Letheby, 2021). According to the author, this leads to two important consequences, which can be dramatically therapeutic for individuals with depression: (i) they can experience a new subjective awareness which is not consistent – and was actually impeded by – previously entrenched beliefs and (ii) those beliefs lose their phenomenal transparency and become opaque (subjects understand that their ideas about themselves are just narratives, not reality itself) (Letheby, 2021). The author argues that an intense psychedelic state can provide direct access to an experiential knowledge about the flexibility of self-representation and about the vast potential of one's mind: experiencing the psychological insight that our narrative self is precisely a narrative – and, as all narratives, may be modified – is the fundamental therapeutic mechanism in psychedelic-assisted therapy.

**Figure 1.**
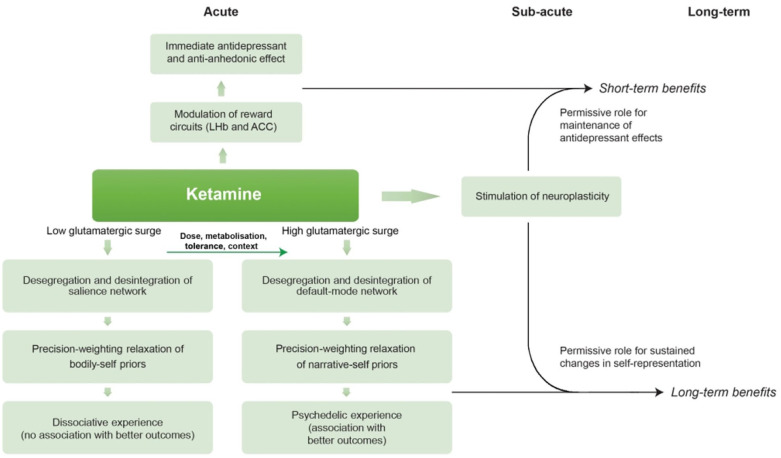
Unified model of ketamine’s properties. Ketamine modulation of reward centres causes immediate short-term antidepressant effects which are maintained by the sub-acute increase in neuroplasticity. Depending on the dose, metabolization rate, previous exposure and context, ketamine can modify synaptic gain to cause a transitory *dissociative* state by inducing a relaxation of the precision weighting of ‘bodily’ self-representation priors encoded in SN nodes or a transitory *psychedelic* state by inducing a relaxation of the precision weighting of ‘narrative’ self-representation priors encoded in DMN nodes. The sub-acute stimulation of neuroplasticity has a permissive role for maintaining changes in self-representation which may confer long-lasting benefits. ACC: anterior cingulate cortex; DMN: default-mode network; LHb: lateral habenula nucleus; SN: salience network.

Another fundamental proposal of Letheby and Gerrans (2017) comes from the early clinical recognition that different aspects of the self-model might be affected to varying extents and in different times during a psychedelic experience, with changes ‘in the body’ preceding changes ‘in the mind’ (Grof, 1980; Masters and Houston, 1966; Savage, 1955). This typical temporal sequence with classic psychedelics, from blurring of body boundaries and loss of sense of ownership for body parts through to later loss of sense of ownership for thoughts, is in agreement with the multi-layered architecture of the self-model as a complex, hierarchical representation of an object or entity having various attributes: spatiotemporal location, a history, personality traits, ownership of a body, and ownership and authorship of thoughts, feelings, and actions (Sui and Humphreys, 2015). Importantly, it may also explain why some studies have found that ego dissolution correlates with modifications in the DMN and others have found correlations with the SN. These authors proposed that psychedelic-induced disruption to SN and DMN integrity should be selectively associated with disruption to embodied and narrative aspects of self-consciousness, respectively (Letheby and Gerrans, 2017). Combining the REBUS model and the predictive self-binding theory with the evidence from ketamine’s research, we propose that:

- In low doses (~0.25–0.5 mg/kg) and with low contextual influence, ketamine may induce desegregation and disintegration of the SN, relaxing priors about bodily self-experience, accounting for its ‘dissociative’ effects.
- In slightly higher doses (0.5–1 mg/kg), ketamine may induce context-dependent modifications at the highest level in the cerebral hierarchy – the DMN – relaxing priors that represent narrative self-experience, accounting for its ‘psychedelic’ effects (Figure 1).

At higher/anaesthetic (~5 mg/kg) doses, ketamine causes slow-delta oscillations that alternate with gamma oscillations (an effect known as gamma burst) inducing a more profound disturbance of large-scale functional networks and a full-blown disruption of frontoparietal communication which is not compatible with conscious awareness, causing unconsciousness (Akeju et al., 2016; Lee et al., 2013).

The linear distribution of NMDA receptors across the cortex (Conti et al., 1994, 1997; Huntley et al., 1994a, 1994b), without an increased density in the visual areas as 5HT2A receptors (Watakabe et al., 2009; Beliveau et al., 2017), explains the lower predominance of visual effects in the ketamine subjective experience, in contrast with classic/serotonergic psychedelics. In terms of the neurobiological organization of the brain, it is sensible that very low doses are enough to disrupt activity in the SN (which is composed of a lower number of areas) and slightly higher doses are needed to disrupt activity in the highest levels of the cerebral hierarchy (as the DMN is composed of a higher number of areas). However, similar to all substances which can cause psychedelic states, the result is highly dependent on the context of administration. Converging evidence suggests the quality of a psychedelic experience – either induced by a serotonergic drug, non-serotonergic drug, meditation or other practises – is heavily influenced by context, including mindset, expectations and environmental factors (Carhart-Harris et al., 2018; Johnson et al., 2008; Smigielski et al., 2019; Studerus et al., 2012). Traditionally, altered states of consciousness were embedded in indigenous rituals and contemplative practices (Nichols, 2004). It has been shown that having clear intentions and expectations before the administration of classic psychedelics is more likely to lead to a peak or mystical-like experience (Haijen et al., 2018). We thus propose that both dosage and context have an important role in shaping the occurrence of a psychedelic-like state under ketamine (Figure 1).

Our model is in line with the psychometric (quantitative) and phenomenological (qualitative) psychological data produced in recent studies and also with anecdotal evidence from the groups which have used ketamine for PAP since the 1970s (Dore et al., 2019; Kolp et al., 2014; Krupitsky and Grinenko, 1997). According to Kolp and colleagues, ketamine may induce four different types of subjective experiences which are dependent on dose, set and setting. They describe an ‘empathogenic experience’, typically attained at doses in the 0.25–0.5 mg/kg IM range, which consists of slight modifications in awareness of the body, with reduced ego defences and feelings of comfort and relaxation; an ‘out-of-body experience’, typically attained at doses in the 0.75–1.5 mg/kg range, which consists in the experience of complete separation from one’s body and significantly diminished ego defences; a ‘near-death experience’, typically attained at doses in the 2.0–3.0 mg/kg IM range, which consists of feeling of departure from one’s body, ego dissolution, experience of physical (body) or psychological (ego) death; and an ‘ego-dissolving transcendental experience’, typically attained at doses in the 2.0–3.0 mg/kg IM range, which consists of an ecstatic state of complete dissolution of the boundaries between the self and external reality, transcendence of time and space and sense of sacredness. We propose that the ‘empathogenic’ and ‘out-of-body’ experiences are caused by slight or intense, respectively, modular disintegration of the SN functional connectivity which reduces the precision of top-down predictions about bodily self-experience while the ‘near-death’ and ‘ego-dissolving transcendental experiences’ are caused by slight or intense, respectively, modular disintegration of the DMN functional connectivity, which reduces the precision of top-down predictions about narrative self-experience.

Other important factors which may influence the acute psychological experience are metabolization rate and previous exposure to the substance. Ketamine undergoes substantial metabolism, initially via nitrogen demethylation to norketamine, a reaction that is catalysed primarily by the liver enzymes CYP2B6, CYP3A4 and CYP2C9 (Hijazi and Boulieu, 2002; Zanos et al., 2018). Afterwards, norketamine is further metabolized to hydroxynorketamine and dehydronorketamine. The individual variability in the metabolism of ketamine has been attributed, in part, to differences in the expression of cytochrome P450 liver enzymes (Hijazi and Boulieu, 2002; Zanos et al., 2018). Based on relative clearance, enzyme types can be classified as poor metabolizers, intermediate metabolizers and ultra-rapid metabolizers (Zheng et al., 2017). It has demonstrated that three different and frequent CYP2C9 alleles are associated with increased ketamine clearance (Zheng et al., 2017) – the relationship between these polymorphisms and the acute psychological effects would be interesting. Regarding repeated exposure, recreational ketamine use may induce tolerance to subjective effects (Sassano-Higgins et al., 2016) and ketamine sensitization has been demonstrated in rodents (Trujillo and Heller, 2020). Regarding its clinical use as an antidepressant, there are case reports describing the occurrence of tolerance to ketamine’s antidepressant effect (Bonnet, 2015; Liebrenz et al, 2009). As Vollenweider and Kometer (2010) show, larger doses of ketamine produce ego dissolution experiences more frequently than lower doses (Vollenweider and Kometer, 2010), although to our knowledge the effect of dose increase in the same patient has never been formally tested. These characteristics make it difficult to predict the acute subjective experience for a specific ketamine session on a specific patient. Further research is needed to better clarify the effects of ketamine dose escalation.

Although we have mainly focused on their similarities, there are also important differences between psilocybin and ketamine. Psilocybin seems to produce changes in the sense of narrative self in a more reliable way than ketamine – we hypothesize that this happens partly because of its serotoninergic neuromodulatory profile, producing changes in DMN connectivity profile at a wider dosage window and which last longer than ketamine (as glutamate is the neurotransmitter which causes the desynchronization, not an indirect modulator). Psilocybin also presents a transient mood-enhancement period in healthy individuals, typically known as the ‘psychedelic afterglow’, which is not described with ketamine (Majić et al., 2015). The acute blocking of lateral LHb bursting activity (Pulcu et al., 2021; Yang et al., 2018) seems to be a specific mechanism for ketamine-induced immediate improvement in anhedonia in depressed individuals and we speculate that it may be the trigger to the normalization in frontostriatal connectivity reported by Mkrtchian et al. (2020). However, we propose that ketamine’s capacity to induce changes in the highest levels of the predictive hierarchy of the brain, such as the DMN, allows for a psychedelic state to occur, in which modifications in self-representation can confer long-lasting benefits. Due to its glutamatergic (non-neuromodulatory) molecular target and shorter half-life, ketamine acute subjective effects are shorter in duration and occur in a narrower dosage window than psilocybin. Ketamine also seems to have higher potential to induce experiences which consist solely of modifications in bodily self-awareness, possibly through disruption of regular SN activity, while the longer duration high-dose psilocybin experiences typically possess a similar initial component (changes in body awareness are present in all classical descriptions of the subjective effects of serotonergic psychedelics (Grof, 1980; Masters and Houston, 1966; Savage, 1955), but then quickly and reliably progress to modifications in narrative self-awareness. Interestingly, lower doses of psilocybin only cause modifications in perception (Vollenweider and Kometer, 2010) which are not associated with long-lasting benefits.

From a theoretical perspective, it is interesting to consider the effects on neuroplasticity, in relation to the effects on synaptic gain or efficacy that underwrite the relaxation of precisely held beliefs about the ‘self’. This distinction speaks to a short-term relaxation (i.e. dissolution) of overly precise beliefs about the narrative (or embodied) self that enable alternative hypotheses about the ‘self’ to be explored. Crucially, this exploratory sense making entails belief updating and neuronal activity that drives activity-dependent plasticity – for which increased neuroplasticity plays a permissive role (Figure 1). This kind of belief updating (about the ‘self’) further emphasizes the importance of the ‘setting’ implicit in assisted therapies. There are several useful metaphors for the ensuing synergy. In optimization theory, it can be likened to simulated annealing, in which the process in question is rendered more malleable through heating as it adapts to new configuration (Mehrabian and Lucas, 2006). In theoretical biology, it emerges as selection for selectability; namely, an increase in mutation rates (c.f., neuroplasticity) in response to environmental changes (Kauffman, 1993; Voigt et al., 2001; Woods et al., 2011). In machine learning, a similar kind of synergy emerges in meta-learning or learning to learn (Doya, 2002). In the present context, these theoretical perspectives emphasize the importance of a separation between short-term and long-term effects on inference and learning, respectively, which may be a unique feature of the psychopharmacology we have been unpacking.

This synergy between acute changes in synaptic gain and sub-acute changes in neural plasticity also suggests that complementing the pharmacological treatment with psychotherapy may have additive benefits. Recently, Muscat et al. (2022) proposed an integrative approach that aims to help improving the development of ketamine treatment protocols and clinical outcomes. The authors propose that preparation sessions, which should be based on a psychoeducational-based approach, may allow to increase efficacy and safety as the multiple levels of the beneficial actions of ketamine are extensively discussed. Furthermore, a careful titration of ketamine’s dosage that allows the induction of a psychedelic state with ego dissolution may provide additional benefits. Also, as the quality of the experience seems to play a pivotal role, optimizing the context, with a safe and comfortable environment that encourages relaxation and introspection, and a carefully curated music selection are important factors. According to Muscat et al, adjunctive psychotherapy sessions, their timing, duration, frequency and goals should also be defined according to the most recent findings in research. We would add that a good therapeutic alliance, which under the HPP framework is a result of interpersonal synchrony (Friston and Frith, 2015), should contribute for an increased freedom to explore new hypothesis about the ‘self’ which could be more adaptive – an argument which is in line with the association between therapeutic alliance, emotional breakthrough and mystical experiences (Murphy et al., 2022). Ketamine’s patent has been dropped several years ago and so it is a very inexpensive medicine per se, but increased treatment costs of adjunctive psychotherapy and accessibility are also important topics.

Our hypotheses are fully testable and outline several future directions. Clinical trials should compare the administration of ketamine in the same setting as in psychedelic research with ketamine administration in regular clinical setting in patients with depression. An additional important comparison group would be ketamine infusions with traditional psychotherapy in between sessions. According to our hypothesis, ketamine administration under the PAP model should increase the proportion of patients attaining ego dissolution and reporting changes in self-representation and this should correlate with better and longer-lasting therapeutic outcomes in depressed subjects. In neuroimaging, changes in bodily self-experience (assessed by the CADSS or the ASC disembodiment factor) during ketamine administration should correlate with changes in SN functional connectivity while changes in narrative self-experience (assessed by the MEQ, ego-dissolution inventory, Psychological Insight Questionnaire or ASC ‘oceanic boundlessness’ factor) should correlate with changes in DMN functional connectivity. We hope this manuscript paves the way for more research on KAP, particularly stimulating RCTs which can generate the approval of ketamine as a treatment for TRD and other disorders.
